# Drivers Are More Physically Active Than Non-Drivers in Older Adults

**DOI:** 10.3390/ijerph15061094

**Published:** 2018-05-28

**Authors:** Shiho Amagasa, Noritoshi Fukushima, Hiroyuki Kikuchi, Tomoko Takamiya, Yuko Odagiri, Koichiro Oka, Shigeru Inoue

**Affiliations:** 1 Department of Preventive Medicine and Public Health, Tokyo Medical University, 6-1-1 Shinjuku, Shinjuku-ku, Tokyo 160-8402, Japan; amagasa@tokyo-med.ac.jp (S.A.); fukufuku@tokyo-med.ac.jp (N.F.); kikuchih@tokyo-med.ac.jp (H.K.); takamiya@tokyo-med.ac.jp (T.T.); odagiri@tokyo-med.ac.jp (Y.O.); 2 Faculty of Sport Sciences, Waseda University, 2-579-15 Mikajima, Tokorozawa, Saitama 359-1192, Japan; koka@waseda.jp

**Keywords:** exercise, sedentary lifestyle, accelerometry, aging, mobility, automobiles, epidemiology

## Abstract

Car use has been identified as sedentary behavior, although it may enhance mobility, particularly in the older population. This cross-sectional study aimed to compare the time spent in objectively determined sedentary behavior (SB) and physical activity (PA) between older drivers and non-drivers. Four hundred and fifty Japanese older adults (74.3 ± 2.9 years) who had valid accelerometer data were included. They were asked to respond to a questionnaire and wear an accelerometer (HJA-350IT, Omron Healthcare) on their waist for 7 consecutive days in 2015. To compare activity time between drivers and non-drivers, we calculated estimated means using analysis of covariance, adjusting for sociodemographic, physical, and psychological factors and accelerometer wear time. Compared to non-drivers, drivers engaged in more light-intensity PA (LPA) (drivers: 325.0 vs. non-drivers: 289.0 min/day) and moderate-to-vigorous PA (drivers: 37.5 vs. non-drivers: 30.0 min/day) and less SB (drivers: 493.4 vs. non-drivers: 535.9 min/day) (all *p* < 0.05). After stratification by age, sex, and residential area, larger effect of driving on PA time was found in older-older adults, in men, and in rural residents. Older drivers were found to be more physically active than non-drivers, suggesting more access to outdoor activities or expanding social network.

## 1. Introduction

Car use has been identified as sedentary behavior and a health risk in the adult population [1,2,3,4]. Initially, Morris et al. found that sedentary drivers of London’s double-decker buses who remain sedentary for a large amount of the day had higher risks of cardiovascular disease than the bus conductors who engaged in physically active jobs [3]. Recent longitudinal studies have also shown that frequent and longer car use is associated with higher cardiovascular disease mortality in men [5] and greater weight gain in adults [1]. Several interventions to replace car use with active transport have been conducted [6,7].

However, contrary to the adult population, car use may not be a health risk in the older population. One possibility is that car use enables mobility and freedom and enables older adults to be more physically active throughout the entire day. For older adults in Japan and other developed countries, driving is often the most preferred mode of transportation [8,9]. Previous studies have indicated that car ownership and driving are associated with independence and life satisfaction in older adults [10,11,12,13]. Driving cessation is likely to result in fewer out-of-home activities as they replace outside activities with indoor activities [14].

A previous questionnaire survey suggested that older adults with 1 or fewer cars were less likely than those with ≥2 cars to achieve the recommended levels of moderate-to-vigorous physical activity (MVPA) (i.e., 150 min/week MVPA) [15]. On the other hand, another recent study showed that there was no difference in accelerometer determined MVPA time between drivers and non-drivers in older American adults [16]. However, in these studies, driving status was evaluated based on assumption (e.g., car ownership). Therefore, direct assessment of whether these people drive or not is required to accurately compare physical activity (PA) time between drivers and non-drivers.

Recent studies report that older adults spend the majority of time in sedentary behavior (SB) and light-intensity PA (LPA) [17,18,19], which can be associated with mortality [20,21,22,23], cognitive decline [24], and cardiometabolic risk biomarkers [25,26,27]. To date, it remains unclear as to how daily activity patterns including SB and LPA differ between older drivers and non-drivers. Therefore, the purpose of this study was to compare the time spent in objectively measured activity classified by intensity and duration between drivers and non-drives in community-dwelling older adults. Also, we aimed to determine whether the impact of driving on each activity differs according to sociodemographic characteristics.

## 2. Materials and Methods

### 2.1. Study Design and Data Collection

Questionnaires were mailed to 1210 community-dwelling older adults aged 70–79 years from February through June 2015. They were originally randomly recruited from resident registries of three municipalities in Japan: Bunkyo city (urban) and Fuchu city (suburban) in Tokyo, and Oyama City (rural) in Shizuoka prefecture, and they responded to a population-based survey conducted in 2010. The details of the sampling process for the 2010 survey and the regional characteristics of each area are presented in a previous study [28]. We sent accelerometers and instructions to participants who agreed to wear an accelerometer. Ethical approval was obtained from the Tokyo Medical University Ethics Committee prior to the survey (No. 2898). Written informed consent was obtained from all participants.

### 2.2. Questionnaire Data

Date of birth, sex, and residential area were obtained from the residential registry of each municipality in 2010. A residential registry is a local government database including information on all the residents. Living arrangement (with others/alone), working status (working with income/not working), driving status (frequently/rarely/no driver’s license), self-rated health, and self-rated physical limitation were assessed by the questionnaire survey carried out in 2015. Older adults who frequently drive a car were defined as drivers whereas others were defined as non-drivers in this study, as we focused on the frequency of driving rather than the possession of a driver’s license. Body mass index (BMI) (kg/m^2^) was calculated by self-reported height and weight. Self-rated health was assessed using one question from SF-8 (Japanese version) that asked participants to rate their health. Participants chose the answer that most accurately described their health from a 6-point scale: excellent, very good, good, fair, poor, and very poor, to the question; “Overall, how would you rate your health during the past 4 weeks?” [29]. Self-rated physical limitation was also evaluated using one question from SF-8: not at all, very little, somewhat, quite a lot, could not do physical activities, to the question; “During the past 4 weeks, how much did physical health problems limit your physical activities (such as walking or climbing stairs)?” [29]. The answers were classified into “no” (not at all and very little) and “yes” (somewhat, quite a lot, and could not do physical activities).

### 2.3. Accelerometry

SB and PA were measured using a tri-axial accelerometer, the Active style Pro HJA-350IT (Omron Healthcare, Kyoto, Japan), which provides data comparable to the devices most commonly used in studies conducted in Western countries [30,31]. Its algorithm and validity have been presented in detail elsewhere [32,33,34]. Previous study reports that activity intensity measured by accelerometers used in this study is relatively accurate in older adults [34]. We obtained 60-s epoch data and calculated metabolic equivalents (METs) values using attached analysis software, BI-LINK PROFESSIONAL EDITION Ver1.0 (Omron Healthcare, Kyoto, Japan). METs-based cutoff was used to define each intensity of activities: ≤1.5 METs for SB, 1.6–2.9 METs for LPA, 3.0–5.9 METs for moderate-intensity PA (MPA), ≥6 METs for vigorous-intensity PA (VPA), and ≥3 METs for MVPA [35,36]. Active style Pro does not detect postural information and hence SB was determined by acceleration.

Participants were asked to wear the accelerometer on their waist for 7 consecutive days while awake, only taking it off for water-based activities (e.g., swimming and showering). No acceleration signal detected for longer than 60 consecutive minutes was defined as “non-wear” [37]. Wear time was calculated by subtracting non-wear time from 1440 min. Records from participants wearing the accelerometer for at least 10 h per day were considered valid [38]. Participants with data from at least 4 days were included in the analysis [37].

### 2.4. Statistical Analyses

The Chi-squared test or Fisher’s exact test was performed to compare participant characteristics between those who agreed to wear an accelerometer and those who did not. Descriptive analyses of the time spent in SB, LPA, and MVPA were conducted, stratified by driving status (drivers and non-drivers). Total MVPA was further classified into 2 types according to the duration of MVPA-bout: short-bout MVPA (<10 min) and long-bout MVPA (≥10 min). Long-bout MVPA was defined as 10 or more consecutive minutes above the moderate intensity threshold, with allowance for interruptions of 1 or 2 min below the threshold [37]. Regarding SB, a bout was considered as a specific duration of time in continuous sedentary time in which no interruption was allowed. In this study, we examined ≥30 min SB and ≥60 min SB, considering previous research on associations of sedentary bout durations with health outcomes [39,40,41].

As the multivariate analysis, we used the analysis of covariance (ANCOVA) to compare SB and PA classified by intensity and duration between drivers and non-drivers, adjusting for wear time, age, sex, residential area, working status, living arrangement, BMI, self-rated health, and physical limitation. Distributions of MVPA (total MVPA, short-bout MVPA, and long-bout MVPA) and prolonged SB (≥30 min SB and ≥60 min SB) were positively skewed and therefore the log transformation was applied when models were run. Estimated means and their 95% confidence intervals (CI) were then back-transformed for the purposes of reporting. To estimate the effect of size, partial eta squared (η_p_^2^) was calculated after adjustment for covariates. Analyses were conducted first for the overall sample, and then separately for age group (younger-older adults [70–74 years]/older-older adults [75–79 years]), sex (men/women), or residential area (urban/suburban/rural). As sensitive analyses, models were rerun (1) excluding older adults with physical limitation (i.e., those who chose the answer: somewhat, quite a lot, and could not do physical activities, to the question; “During the past 4 weeks, how much did physical health problems limit your physical activities (such as walking or climbing stairs)?”; (2) stratified by “not at all” of physical limitation or the other; and (3) using the original 3 categories (frequently/rarely/no driver’s license) as exposure variable. All statistical analyses were performed using IBM SPSS Statistics version 21 software (IBM Corp., Tokyo, Japan) and R version 3.3.3. for Mac; a *p*-level of less than 0.05 was considered to indicate a statistically significant difference between groups.

## 3. Results

### 3.1. Participant Characteristics

Of the 1210 people surveyed, 988 older adults returned the questionnaire (response rate: 81.7%), and 478 of those agreed to wear the accelerometer. However, 28 were excluded owing to the following reason: not meeting the wearing time criteria (i.e., wearing at least four days of ≥10 h/day) (*n* = 7), refusal to wear or failure to return the accelerometer (*n* = 15), and system error (*n* = 6). Therefore, a total of 450 older adults (255 men, 56.7%) were included in this study. When participant characteristics were compared between those who agreed to wear an accelerometer and those who did not, significant differences were observed in sex (proportion of agreement; men: 55.6%, women: 47.7%, *p* = 0.015), self-rated health (proportion of agreement; excellent: 78.3%, very good: 66.0%, good: 59.4%, fair: 60.4%, poor: 50.0%, and very poor: 33.3%, *p* = 0.038), and physical limitation (proportion of agreement; not at all: 63.2%, very little: 55.1%, somewhat: 65.5%, quite a lot:54.2%, could not do physical actives: 17.6%, *p* = 0.001). No significant differences were found in driving status (proportion of agreement; drivers: 55.2%, non-drivers: 50.6%, *p* = 0.174).

The mean age of the participants was 74.3 ± 2.9 years. Fewer than half of the participants were drivers (*n* = 209, 46.4%), and most of the study population was living with others and had good self-rated health (Table 1). Most of the older adults categorized as non-drivers did not have driver license (*n* = 172, 71.4%).

### 3.2. Descriptive Data of Accelerometers

Time spent in objectively measured SB and PA in older drivers and non-drivers is presented in Table 2. Mean accelerometer wear time was 865.9 min/day in drivers and 880.8 min/day in non-drivers. Most of the wear time was spent in SB (57.9% in drivers, 61.4% in non-drivers), whereas the median time spent in MVPA was no more than 5% (5.0% in drivers, 4.1% in non-drivers).

### 3.3. Results of Analysis of Covariance

**Figure 1:**
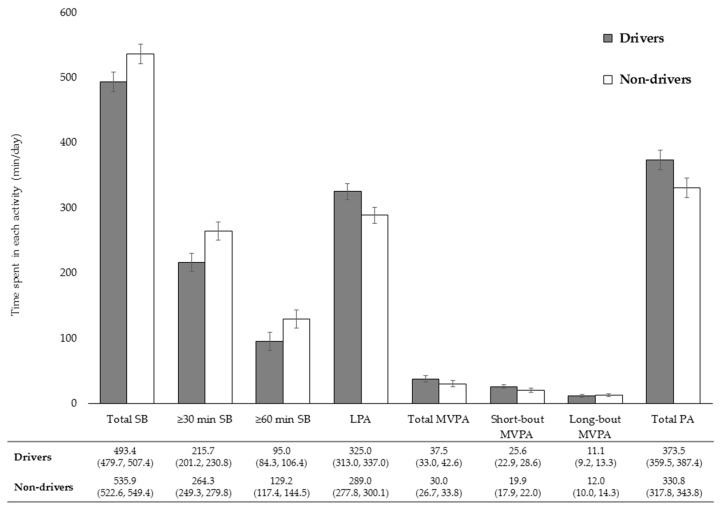
Comparisons of objectively measured sedentary and physical activity time between older drivers and non-drivers. Abbreviations; SB: sedentary behavior, LPA: light-intensity physical activity, MVPA: moderate-to-vigorous physical activity. Short-bout: lasting <10 min, long-bout: lasting ≥10 min. Estimated mean was adjusted for accelerometer wear time, age, gender, residential area, working status, living arrangement, body mass index, self-rated health, and physical limitation. Bars represent 95% CI of the mean. Values below the x-axis are adjusted means (95% CI).

Comparisons of objectively measured SB and PA between drivers and non-drivers after adjustment for covariates are shown in Figure 1. Relative to non-drivers, drivers engaged in 42.7 min more total PA (i.e., LPA + MVPA) (*p* < 0.001) and 42.5 min less SB (*p* < 0.001) on account of 36.0 min more LPA (*p* < 0.001) and 7.5 min more total MVPA (*p* = 0.024). This difference in total MVPA time was mainly a result of not long-bout MVPA (*p* = 0.581) but short-bout MVPA (*p* = 0.003). Drivers engaged in 48.6 min less ≥30 min SB (*p* = 0.001) and less 34.2 min ≥60 min SB (*p* < 0.001).

In sensitivity analyses excluding those with physical limitation, the results are much the same. Differences in SB, ≥30 min SB, ≥60 min SB, LPA, short-bout MVPA, and total PA time between drivers and non-drivers were remained statistically significant, whereas differences in total MVPA time between them were no longer significant. For older adults who chose the answer “not at all” of physical limitation, the differences of each activity time between drivers and non-drivers were not statistically significant (Tables S3 and S4). The effect size of driving on SB and PA was larger in those with any physical limitation compared to those without physical limitation.

In sensitivity analyses using the original driving status categories (frequently/rarely/no driver’s license) as exposure variable, the results did not change (Tables S3 and S4). Older adults who drive a car frequently had the highest level of PA.

Results of stratified analyses of SB and PA are shown in Table 3 and Table 4, respectively. Drivers engaged in less prolonged SB as well as total SB than non-drivers in older-older adults (total SB: 50.9 min, *p* = 0.002; ≥30 min SB: 48.0 min, *p* = 0.010; ≥60 min SB: 36.7 min, *p* = 0.004) and rural residents (total SB: 56.2 min, *p* = 0.002; ≥30 min SB: 43.9 min, *p* = 0.028; ≥60 min SB: 32.0 min, *p* = 0.019). In terms of PA, there were no significant differences in long-bout MVPA between drivers and non-drivers at any groups (younger-older adults: *p* = 0.976, older-older adults: *p* = 0.659, men: *p* = 0.443, women: *p* = 0.828, urban residents: *p* = 0.385, suburban residents: *p* = 0.781, rural resident: *p* = 0.689). On the other hand, drivers accumulated more short-bout MVPA than non-drivers in older-older adults (8.4 min, *p* = 0.004), women (9.7 min, *p* = 0.022), suburban residents (6.9 min, *p* = 0.010), and rural residents (10.4 min, *p* = 0.005). Except for women (*p* = 0.134), drivers engaged in more LPA in all groups (younger-older adults: 27.8 min, *p* = 0.023; older-older adults: 43.2 min, *p* = 0.003; men: 42.8, *p* < 0.001; urban residents: 39.2 min, *p* = 0.033; suburban residents: 29.9 min, *p* = 0.045, rural resident: 41.3 min, *p* = 0.007). Consequently, drivers spent longer time engaging in total PA time than non-drivers in all groups, (younger-older adults: 33.4 min, *p* = 0.023; older-older adults: 50.9 min, *p* = 0.002; men: 45.5, *p* = 0.001; suburban residents: 35.2 min, *p* = 0.037, rural resident: 56.3 min, *p* = 0.002) although no statistically significant differences were found between drivers and non-drivers in women (*p* = 0.050) and in urban residents (*p* = 0.065). The effect size of driving on SB and PA was larger in older-older adults, men, and rural residents, compared to in younger-older adults, women, and urban and suburban residents, respectively.

## 4. Discussion

Our data provide new information on regarding the potential effect of driving status on detailed daily activity patterns in Japanese older adults. In summary, we found that older drivers were more physically active than non-drivers regarding total daily PA levels. These findings did not change after stratification by age, gender, and residential area, although no statistically significant differences were found between drivers and non-drivers in women and in urban residents.

The underlying reason older drivers are more active than older non-drivers may be enhanced mobility. Private car use may increase the mobility of older adults and make social inclusion and outside activities less difficult [42]. A previous study suggested that driving cessation was strongly associated with a decline in the amount of out-of-home activities [42]. Social inclusion among older adults, for example, is linked to more MVPA time [43,44] and less sedentary time [43]. In this study, a greater difference in MVPA time between drivers and non-drivers were found in rural residents compared with urban and suburban residents. This is probably because rural residents are more likely to rely on their private car for daily mobility [9].

Considering the accumulated line of evidence on the associations of SB and PA with health benefits [20,21,22,45,46,47,48,49], differences in the time spent in SB and PA between drivers and non-drivers may be significant. In this study, drivers had approximately 42 min shorter SB, 36 min longer LPA, and 8 min longer total MVPA, which were mainly owing to short-bout MVPA. A recent study, for example, showed that a greater PA was associated with lower all-cause mortality risk, estimating that replacing 30 min of SB with LPA reduces mortality risk (after 5 years of follow-up: hazard ratio [HR] = 0.80, 95% CI = 0.75–0.85) in adults age 50–79 years [45]. Another study found that replacing 10 min of SB with the same amount of MPA resulted in a significantly lower odds ratio (OR) for metabolic syndrome prevalence (OR = 0.89, 95% CI = 0.82–0.97), high waist circumference (OR = 0.94, 95% CI = 0.88–0.99), high triglyceride levels (OR = 0.91, 95% CI = 0.83–0.99), and high blood pressure (OR = 0.92, 95% CI = 0.85–0.99) [49]. Given these findings, drivers may be received more health benefits by PA than non-drivers, especially in older-older adults, in men, and in rural residents.

In this study, older drivers are more physically active than older non-drivers. Although it has been considered that car use accelerates sedentary lifestyle, older adults who drive a car for transportation do not always engage in more SB and less PA throughout the entire day. Findings of this study may suggest that policy and intervention which allow older adults to keep driving and mobility are effective to promote PA levels, independence, and health.

This study has several limitations to be considered. First, this is a cross-sectional design and there may be reverse causality. It may be that those people who are more active drive more. Second, physical performance and cognitive function can be an important confounding factors in our analyses. Although we adjusted for self-rated health and physical limitation as potential confounders, residual confounding needs to be considered for interpretation of these results. Third, an accelerometer cannot accurately detect postural information (i.e., sitting and standing) and using MET cut-points to define activity intensities may misclassify activity level for older adults. Besides, some types of activities such as swimming and cycling may be underestimated. Fourth, this study did not analyze driving time and consider the purpose for driving, which may affect a driver’s SB and PA. Fifth, our sample was limited to older adults aged 70–79 years. Further investigation including a wider range of the older population is warranted. Finally, the 3 locations included in the present study may not fully represent all rural, suburban, and urban cities. Although these cities were chosen based on their urbanization levels, it is necessary to consider the generalizability of these findings.

## 5. Conclusions

This study compared the time spent in objectively assessed SB and PA between drivers and non-drivers of community-dwelling older adults in Japan. Older drivers were found to be more physically active, suggesting more access to outdoor activities or expanding social network. The effect of driving on PA time was larger in older-older adults, men, and rural residents, compared to younger-older adults, women, and urban residents, respectively. Longitudinal studies are needed to verify directionality to make the case for physical activity policy and intervention effectiveness.

## Figures and Tables

**Table 1:** Participant characteristics.

Variables	Drivers (*n* = 209)	Non-Drivers (*n* = 241)	
	*n* (%)	*n* (%)	*p*-Value
Sex			**<0.001** ^a^
Men	160 (76.6)	95 (39.4)	
Women	49 (23.4)	146 (60.6)	
Age			0.999 ^a^
70–74 years	109 (52.2)	125 (51.9)	
75–79 years	100 (47.8)	116 (48.1)	
Residential area			**<0.001** ^a^
Urban	28 (13.4)	114 (47.3)	
Suburban	59 (28.2)	83 (34.4)	
Rural	122 (58.4)	44 (18.3)	
Living arrangement			**0.006** ^a^
With others	189 (38.3)	197 (21.3)	
Alone	19 (61.7)	44 (78.7)	
(missing *n* = 1)			
Working with income			**<0.001** ^a^
Working	80 (90.9)	51 (81.7)	
Not working	129 (9.1)	188 (18.3)	
(missing *n* = 2)			
Body mass index (BMI)			0.140 ^a^
<25.0 kg/m^2^	165 (78.9)	204 (84.6)	
≥25.0 kg/m^2^	44 (21.1)	37 (15.4)	
Self-rated health			**0.046** ^b^
Excellent	9 (4.3)	8 (3.3)	
Very good	50 (24.0)	49 (20.5)	
Good	121 (58.2)	126 (52.7)	
Fair	26 (12.5)	42 (17.6)	
Poor	2 (1.0)	11 (4.6)	
Very poor	0 (0.0)	3 (1.3)	
(missing *n* = 3)			
Self-rated physical limitation			0.206 ^b^
Not at all	127 (61.1)	125 (53.0)	
Very little	41 (19.7)	55 (23.3)	
Somewhat	35 (16.8)	42 (17.8)	
Quite a lot	5 (2.4)	11 (4.7)	
Could not do physical activities	0 (0.0)	3 (1.3)	
(missing *n* = 6)			
Moderate to vigorous physical activity			0.594 ^a^
<150 min/week (not meeting guidelines)	150 (71.8)	179 (74.3)	
≥150 min/week (meeting guidelines)	59 (28.2)	62 (25.7)	

*p*-values were calculated by the Chi-squared test ^a^ or Fisher’s exact test ^b^ bold indicates statistical significance (*p* < 0.05).

**Table 2:** Unadjusted data of objectively measured activity time (min/day) in older drivers and non-drivers.

Variables	Drivers (*n* = 209)			Non-Drivers (*n* = 241)		
	Mean ± SD	Median	(25%, 75%)	Mean ± SD	Median	(25%, 75%)
Wear time	865.9 ± 86.8	875.6	(806.3, 923.1)	880.8 ± 93.1	876.1	(819.3, 941.8)
SB	501.5 ± 118.1	508.1	(416.4, 580.3)	539.3 ± 118.1	530.4	(449.6, 608.8)
LPA	315.0 ± 96.8	303.3	(244.0, 374.2)	300.7 ± 106.1	302.5	(222.4, 371.5)
Total MVPA	49.4 ± 33.0	42.6	(23.4, 65.7)	40.7 ± 29.2	34.8	(19.0, 59.0)
Short-bout MVPA	33.0 ± 22.1	27.6	(16.8, 43.3)	25.8 ± 17.9	21.9	(12.7, 33.2)
Long-bout MVPA	16.4 ± 20.4	9.1	(2.0, 22.9)	14.9 ± 18.2	7.8	(1.2, 21.7)

Abbreviations; SB: sedentary behavior, LPA: light-intensity physical activity, MVPA: moderate-to-vigorous physical activity. Short-bout: lasting <10 min, long-bout: lasting ≥10 min.

**Table 3:** Comparisons of objectively measured sedentary time (min/day) between older drivers and non-drivers by sociodemographic characteristics.

Independent Variables	Total SB				≥30 min SB				≥60 min SB			
	EM	(95% CI)	η_p_^2^	*p*-Value	EM	(95% CI)	η_p_^2^	*p*-Value	EM	(95% CI)	η_p_^2^	*p*-Value
*Stratified by age group* ^a^												
Younger-older adults (70–74 years)			0.023	**0.023**			0.020	**0.034**			0.012	0.113
Drivers	507.4	(488.4, 526.5)			216.8	(198.6, 236.6)			94.8	(82.0, 109.6)		
Non-drivers	540.8	(523.2, 558.4)			250.0	(230.7, 271.0)			113.0	(99.1, 129.1)		
Older-older adults (75–79 years)			0.048	**0.002**			0.033	**0.010**			0.043	**0.004**
Drivers	493.2	(472.3, 514.2)			194.1	(173.8, 216.8)			80.5	(68.4, 95.1)		
Non-drivers	544.1	(524.5, 563.8)			242.1	(217.8, 268.5)			117.2	(100.0, 137.1)		
*Stratified by sex* ^b^												
Men			0.049	**0.001**			0.052	**<0.001**			0.049	**0.001**
Drivers	532.0	(517.8, 546.3)			233.9	(217.8, 250.6)			98.6	(87.9, 110.7)		
Non-drivers	577.5	(558.3, 596.7)			293.8	(267.9, 322.8)			141.9	(121.6, 165.6)		
Women			0.022	0.050			0.007	0.272			0.004	0.392
Drivers	459.9	(429.2, 490.7)			178.2	(152.4, 208.9)			79.6	(63.0, 100.5)		
Non-drivers	497.3	(480.6, 513.9)			198.6	(182.4, 215.8)			90.2	(79.3, 102.3)		
*Stratified by residential area* ^c^												
Urban			0.027	0.065			0.028	0.059			0.032	**0.049**
Drivers	528.2	(491.9, 564.4)			211.8	(177.8, 252.3)			80.5	(58.6, 110.7)		
Non-drivers	567.4	(550.2, 584.5)			257.0	(236.6, 279.9)			116.1	(100.0, 134.9)		
Suburban			0.033	**0.037**			0.025	0.073			0.014	0.180
Drivers	523.1	(499.2, 547.0)			221.8	(198.2, 248.9)			97.7	(82.0, 116.4)		
Non-drivers	558.3	(538.6, 578.0)			256.4	(233.3, 281.8)			115.3	(99.8, 133.0)		
Rural			0.058	**0.002**			0.031	**0.028**			0.035	**0.019**
Drivers	460.0	(442.8, 477.2)			188.4	(172.2, 206.1)			83.6	(73.5, 95.1)		
Non-drivers	516.2	(486.2, 546.3)			232.3	(198.6, 272.3)			115.6	(92.3, 145.2)		

^a^ Adjusted for wear time, age, sex, residential area, working status, living arrangement, body mass index, self-rated health, and physical limitation; ^b^ Adjusted for wear time, age, residential area, working status, living arrangement, body mass index, self-rated health, and physical limitation; ^c^ Adjusted for wear time, age, sex, working status, living arrangement, body mass index, self-rated health, and physical limitation; Abbreviations; SB: sedentary behavior, EM: estimated mean, CI: confidence interval. Bold indicates statistical significance (*p* < 0.05).

**Table 4:** Comparisons of objectively measured physical activity time (min/day) between older drivers and non-drivers by sociodemographic characteristics.

Independent Variables	LPA				Total MVPA				Short-bout MVPA				Long-bout MVPA				Total PA (LPA + MVPA)			
	EM	(95% CI)	η_p_^2^	*p*-Value	EM	(95% CI)	η_p_^2^	*p*-Value	EM	(95% CI)	η_p_^2^	*p*-Value	EM	(95% CI)	η_p_^2^	*p*-Value	EM	(95% CI)	η_p_^2^	*p*-Value
*Stratified by age group* ^a^																				
Younger-older adults (70–74 years)			0.023	**0.023**			0.003	0.397			0.006	0.271			0.000	0.976			0.023	**0.023**
Drivers	318.1	(302.2, 334.0)			36.3	(30.3, 43.5)			24.3	(20.8, 28.3)			12.3	(9.4, 16.1)			367.1	(348.0, 386.1)		
Non-drivers	290.3	(275.7, 305.0)			32.3	(27.4, 38.1)			21.3	(18.5, 24.5)			12.2	(9.4, 16.0)			333.7	(316.1, 351.3)		
Older-older adults (75–79 years)			0.045	**0.003**			0.025	**0.026**			0.042	**0.004**			0.001	0.659			0.048	**0.002**
Drivers	331.6	(313.1, 350.1)			38.3	(31.9, 45.9)			26.9	(22.8, 31.8)			10.3	(8.0, 13.3)			379.4	(358.4, 400.4)		
Non-drivers	288.4	(271.0, 305.8)			28.0	(23.6, 33.2)			18.5	(15.8, 21.6)			11.3	(8.8, 14.4)			328.5	(308.8, 348.2)		
*Stratified by sex* ^b^																				
Men			0.057	**<0.001**			0.009	0.140			0.015	0.055			0.003	0.443			0.049	**0.001**
Drivers	277.9	(265.5, 290.3)			34.6	(30.2, 39.6)			21.5	(19.1, 24.2)			11.6	(9.5, 14.2)			322.4	(308.1, 336.7)		
Non-drivers	235.1	(218.4, 251.8)			28.8	(24.0, 34.6)			17.5	(14.9, 20.5)			13.4	(10.1, 17.7)			276.9	(257.8, 296.1)		
Women			0.013	0.134			0.017	**0.079**			0.029	**0.022**			0.000	0.828			0.022	0.050
Drivers	382.7	(356.8, 408.7)			43.0	(33.0, 56.0)			33.6	(26.5, 42.6)			11.0	(7.5, 16.3)			439.4	(408.6, 470.1)		
Non-drivers	358.7	(344.6, 372.7)			32.2	(27.9, 37.2)			23.9	(21.0, 27.2)			10.4	(8.5, 12.9)			402.0	(385.4, 418.7)		
*Stratified by residential area* ^c^																				
Urban			0.036	**0.033**			0.001	0.777			0.000	0.939			0.008	0.385			0.027	0.065
Drivers	304.9	(273.6, 336.2)			31.3	(21.8, 44.9)			19.1	(13.9, 26.4)			10.7	(6.9, 16.6)			347.1	(310.9, 383.4)		
Non-drivers	265.7	(250.9, 280.5)			29.5	(24.9, 35.0)			18.9	(16.2, 21.9)			13.4	(10.8, 16.6)			307.9	(290.8, 325.1)		
Suburban			0.031	**0.045**			0.027	0.063			0.050	**0.010**			0.001	0.781			0.033	**0.037**
Drivers	308.5	(287.4, 329.6)			34.9	(27.9, 43.7)			22.9	(18.8, 27.8)			11.2	(7.8, 16.1)			350.1	(326.2, 374.0)		
Non-drivers	278.6	(261.1, 296.0)			26.0	(21.6, 31.3)			16.0	(13.6, 18.7)			12.1	(8.9, 16.3)			314.9	(295.2, 334.6)		
Rural			0.045	**0.007**			0.031	**0.027**			0.051	**0.005**			0.001	0.689			0.058	**0.002**
Drivers	356.0	(341.6, 370.5)			44.4	(38.5, 51.2)			33.3	(29.4, 37.6)			10.8	(8.6, 13.6)			412.6	(395.3, 429.8)		
Non-drivers	314.7	(289.5, 339.8)			31.7	(24.7, 40.6)			22.9	(18.5, 28.4)			9.8	(6.5, 14.8)			356.3	(326.3, 386.4)		

^a^ Adjusted for wear time, age, sex, residential area, working status, living arrangement, body mass index, self-rated health, and physical limitation; ^b^ Adjusted for wear time, age, residential area, working status, living arrangement, body mass index, self-rated health, and physical limitation; ^c^ Adjusted for wear time, age, sex, working status, living arrangement, body mass index, self-rated health, and physical limitation; Abbreviations; LPA: light-intensity physical activity, MVPA: moderate-to-vigorous physical activity, PA: physical activity, EM: estimated mean, CI: confidence interval; Short-bout: lasting <10 min, long-bout: lasting ≥10 min. Bold indicates statistical significance (*p* < 0.05).

## Supplementary Materials

The following are available online at http://www.mdpi.com/1660-4601/15/6/1094/s1. Table S1: Comparisons of objectively measured sedentary time (min/day) by driving status (frequently/rarely/no driver’s license). Table S2: Comparisons of objectively measured physical activity time (min/day) by driving status (frequently/rarely/no driver’s license). Table S3: Comparisons of objectively measured sedentary time (min/day) by physical limitation status. Table S4: Comparisons of objectively measured physical activity time (min/day) by physical limitation status.
