# Supplementary material for: Drivers Are More Physically Active Than Non-Drivers in Older Adults
Source: Int J Environ Res Public Health. 2018 May 28;15(6):1094. doi: 10.3390/ijerph15061094 (PMC6025007; doi:10.3390/ijerph15061094)
Supplement: Supplementary file 1 [file ijerph-15-01094-s001.pdf]

International Journal of
Environmental Research and Public Health

MDPI

Supplementary Tables

S1. Comparisons of objectively measured sedentary time (min/day) by driving status

Independent variables	Total SB			≥ 30-min SB			≥ 60-min SB		
	EM	(95% CI)	P-value	EM	(95% CI)	P-value	EM	(95% CI)	P-value
Driving status			**<0.001**			**0.003**			**0.002**
Frequently	500.5	(486.5, 514.5)		205.1	(191.4, 220.3)		87.9	(78.7, 97.9)	
Rarely	539.3	(517.1, 561.6)		247.2	(220.8, 276.1)		125.9	(105.4, 150.3)	
No driver's license	544.8	(529.2, 560.3)		247.2	(229.1, 267.3)		111.2	(98.4, 125.3)	

Estimated mean was adjusted for wear time, age, sex, residential area, working status, living arrangement, body mass index, self-rated health, and physical limitation. Abbreviations; SB: sedentary behavior, EM: estimated mean, CI: confidence interval. Bold indicates statistical significance (P<0.05).

S2. Comparisons of objectively measured physical activity time (min/day) by driving status

Independent variables	LPA			Total MVPA			Short-bout MVPA			Long-bout MVPA			Total PA (LPA + MVPA)		
	EM	(95% CI)	P-value	EM	(95% CI)	P-value	EM	(95% CI)	P-value	EM	(95% CI)	P-value	EM	(95% CI)	P-value
Driving status			**0.001**			**0.001**			**<0.001**			0.774			**<0.001**
Frequently	324.7	(312.7, 336.8)		37.8	(33.3, 43.0)		25.8	(23.1, 28.8)		11.1	(9.2, 13.3)		373.5	(359.5, 387.5)	
Rarely	284.4	(265.3, 303.6)		38.5	(31.4, 47.1)		24.6	(20.6, 29.4)		12.6	(9.5, 16.8)		334.6	(312.4, 356.9)	
No driver's license	291.3	(277.9, 304.7)		26.7	(23.2, 30.8)		17.9	(15.8, 20.3)		11.8	(9.4, 14.7)		329.2	(313.6, 344.8)	

Estimated mean was adjusted for wear time, age, sex, residential area, working status, living arrangement, body mass index, self-rated health, and physical limitation. Abbreviations; LPA: light-intensity physical activity, MVPA: moderate-to-vigorous physical activity, PA: physical activity, EM: estimated mean, CI: confidence interval. Short-bout: lasting <10 minutes, long-bout: lasting ≥10 minutes. Bold indicates statistical significance (P<0.05).

S3. Comparisons of objectively measured sedentary time (min/day) by physical limitation status.

Independent variables	Total SB				≥ 30-min SB				≥ 60-min SB			
	EM	(95% CI)	η_P^2	P-value	EM	(95% CI)	η_P^2	P-value	EM	(95% CI)	η_P^2	P-value
Not at all			0.006	0.216			0.003	0.397			0.005	0.276
Drivers	513.3	(496.1, 530.5)			219.8	(202.3, 239.3)			96.6	(84.5, 110.4)		
Non-drivers	530.5	(513.1, 547.8)			232.8	(214.3, 253.5)			108.6	(94.8, 124.2)		
With any limitation			0.091	**<0.001**			0.076	**<0.001**			0.064	**0.001**
Drivers	482.8	(459.3, 506.3)			187.9	(167.1, 210.9)			76.7	(64.1, 92.0)		
Non-drivers	554.5	(534.9, 574.2)			259.4	(235.5, 286.4)			121.1	(104.0, 140.9)		

Estimated mean was adjusted for wear time, age, sex, residential area, working status, living arrangement, body mass index, and self-rated health. Abbreviations; SB: sedentary behavior, EM: estimated mean, CI: confidence interval. Bold indicates statistical significance (P<0.05).

S4. Comparisons of objectively measured physical activity time (min/day) by physical limitation status.

Independent variables	LPA				Total MVPA				Short-bout MVPA				Long-bout MVPA				Total PA (LPA + MVPA)			
	EM	(95% CI)	η_P^2	P-value	EM	(95% CI)	η_P^2	P-value	EM	(95% CI)	η_P^2	P-value	EM	(95% CI)	η_P^2	P-value	EM	(95% CI)	η_P^2	P-value
Not at all			0.007	0.187			0.001	0.641			0.001	0.681			<0.001	0.986			0.006	0.216
Drivers	313.2	(298.5, 327.8)			41.0	(35.5, 47.5)			25.8	(22.6, 29.4)			13.1	(10.4, 16.6)			364.5	(347.3, 381.7)		
Non-drivers	297.6	(282.8, 312.4)			38.8	(33.5, 45.0)			24.7	(21.7, 28.2)			13.2	(10.4, 16.7)			347.4	(330.0, 364.7)		
With any limitation			0.081	**<0.001**			0.044	**0.005**			0.072	**<0.001**			0.004	0.465			0.091	**<0.001**
Drivers	340.0	(319.7, 360.4)			34.3	(27.4, 42.9)			25.8	(21.2, 31.4)			8.6	(6.5, 11.5)			385.3	(361.8, 408.8)		

Non-drivers	281.8	(264.7, 298.8)	21.6	(17.9, 26.0)	15.2	(12.9, 18.0)	10.1	(7.8, 13.1)	313.5	(293.9, 333.2)

Estimated mean was adjusted for wear time, age, sex, residential area, working status, living arrangement, body mass index, and self-rated health. Abbreviations; LPA: light-intensity physical activity, MVPA: moderate-to-vigorous physical activity, PA: physical activity, EM: estimated mean, CI: confidence interval. Short-bout: lasting <10 minutes, long-bout: lasting ≥10 minutes. Bold indicates statistical significance ($P<0.05$).

© 2018 by the authors. Submitted for possible open access publication under the terms and conditions of the Creative Commons Attribution (CC BY) license (http://creativecommons.org/licenses/by/4.0/).
